# Population Size Estimation of People Who Use Illicit Drugs and Alcohol in Iran (2015-2016)

**DOI:** 10.34172/ijhpm.2022.6578

**Published:** 2022-09-10

**Authors:** Azam Rastegari, Mohammad Reza Baneshi, Ahmad Hajebi, Alireza Noroozi, Mohammad Karamouzian, Mostafa Shokoohi, Ali Mirzazadeh, Toktam Khojasteh Bojnourdi, Naser Nasiri, Saiedeh Haji Maghsoudi, Ali Akbar Haghdoost, Hamid Sharifi

**Affiliations:** ^1^Modeling in Health Research Center, Institute for Futures Studies in Health, Kerman University of Medical Sciences, Kerman, Iran.; ^2^Center for Longitudinal and Life Course Research, School of Public Health, Faculty of Medicine, The University of Queensland, Brisbane, QLD, Australia.; ^3^Research Center for Addiction & Risky Behaviors (ReCARB), Psychosocial Health Research Institute, Iran University of Medical Sciences, Tehran, Iran.; ^4^Department of Psychiatry, Faculty of Medicine, Iran University of Medical Sciences, Tehran, Iran.; ^5^Iranian National Center for Addiction Studies, Tehran University of Medical Sciences, Tehran, Iran.; ^6^Centre On Drug Policy Evaluation, St. Michael’s Hospital, Toronto, ON, Canada.; ^7^HIV/STI Surveillance Research Center, and WHO Collaborating Center for HIV, Kerman University of Medical Sciences, Kerman, Iran.; ^8^Institute for Health Policy Studies, University of California, San Francisco, CA, USA.; ^9^Ministry of Health and Medical Education, Tehran, Iran.

**Keywords:** Hidden Groups, Illicit Drug, Network Scale-Up, Alcohol, Iran

## Abstract

**Background:** Estimating the number of people using illicit drugs and alcohol is necessary for informing health policy and programming. However, it is often challenging to reliably estimate the size of these marginalized populations through direct methods. In this study, we estimated the population size of these groups using the indirect Network Scale-Up (NSU) method in Iran from 2015 to 2016.

**Methods:** Using a self-administered questionnaire, we asked 15 124 individuals (54% men) about the number of people they know who used different types of drugs at least once in the past 12 months. Prevalence estimates were reported per 100 000 population. The uncertainty level (UL) was calculated using the bootstrap method.

**Results:** The average age of the respondents was 33 years old, and 35.1% of them were unmarried. The most common drugs and their prevalence were as follows: opium (2534 [95% UL: 2467-2598]), hashish (849 [95% UL: 811-886]), stimulants (methamphetamine, ecstasy pills, cocaine, and Ritalin) (842 [95% UL: 802-879]), heroin/crack (578 [95% UL: 550-607]), and drug injection (459 [95% UL: 438-484]). Additionally, we estimated the prevalence of alcohol use as 2797 (95% UL: 2731-2861). On average, substance use was 5.23 times more prevalent among men than women. Opium use was more prevalent among individuals aged >50 years old. Moreover, alcohol use was more prevalent among participants between 18 and 30 years old (5164 per 100 000 population).

**Conclusion:** Although opium continues to be the most prevalent illicit drug in Iran, the patterns of illicit drug use are heterogeneous among different age groups, genders, and provinces. Age-gender specific and culturally appropriate interventions are warranted to meet the needs of people in different subgroups.

## Background

**Key Messages**

** Implications for policy makers**

- Given the high prevalence of substance and alcohol use among young people, creating appropriate facilities in the cultural, economic, and social fields for this group seems necessary.
- Development of general policies for the prevention of alcohol and narcotics use in the country based on the estimates obtained from this article.
- Reconsideration of the support programs for high-risk groups for human immunodeficiency virus (HIV).
- Identify high-risk provinces and develop appropriate control programs with each province.
- Allocate the necessary financial resources to prevent and provide prevention programs for drug and alcohol consumers.

** Implications for the public**

This study suggests that the prevalence of drug, stimulant and alcohol use in the country is increasing. The prevalence is higher among young people, and the tendency to use synthetic drugs has increased. Although drug use was more prevalent among men, the proportions of synthetic drugs and alcohol use among women is alarming. Considering age, sex, and geographical variations, appropriate intervention programs should be designed to reduce the burden of drug use in the country.

Substance use disorders are global public health concerns that have led to social, mental, and physical harms across several communities. Globally, in 2016, 31.8 million (95% confidence interval [CI]: 27.4, 36.6) disability-adjusted life-years (DALYs) and 1.3% (95% CI: 1.2, 1.5) of all DALYs were reportedly due to drug use.^1,2^ On average, around 3 million people annually die due to the consequences of alcohol use disorder. Moreover, the burden of diseases attributable to alcohol use was calculated as about 5%.^3^

 Substance use disorders are the third most significant public health concern in Iran.^4,5^ Almost 2% of all diseases in Iran are due to substance use, including alcohol and illicit drugs.^4^ The United Nations Office of Drug and Crimes has announced that Iran is among the countries where the use of heroin, cannabis, and methamphetamine is considerably high.^4^ Correspondingly, this reflects the ease of drug accessibility in Iran, which may be partially related torelated to sharing a long border with Afghanistan (a major producer of opium worldwide) and being in the trafficking paths of transferring drugs to Europe (eg, the Balkan route). The population of people who use drugs has continuously increased in the past few decades, and it has been shown that young people are more engaged in substance use practices.^6^

 Moreover, the use of newer drugs, such as hashish (cannabis that is collected from the compressed resin glands, called trichomes, which is stronger than the substances obtained from the buds and leaves of this plant)^7^ and methamphetamine, has increased over time and contributed to human immunodeficiency virus (HIV) epidemics. The DALYs and death rates attributable to alcohol and drug use have significantly increased among all age groups and both genders in Iran. For example, the DALY rate attributable to drug use disorders among men has increased from 399.6/100 000 in 1990 to 804.5/100 000 in 2010.^4^

 Estimating the number of people who use drugs or alcohol is crucial to explore the trends of use over time, to investigate the usefulness of health policies, and to prioritize national plans. Due to stigma and the legal prohibition of drug use in some countries, especially in Iran, studying the prevalence and trend of drug use requires special measures. The Network Scale-Up (NSU) method provides researchers with a simple, relatively inexpensive, and powerful indirect tool for size estimation of marginalized groups. The classic idea behind the NSU method assumes that the prevalence of a behavior in the network of a sample is more or less the same as that in the general population. For example, the proportion of cancer patients in the network of a sample of respondents is comparable with the prevalence of cancer in the population. The NSU is an indirect method in which members of the general population are asked about the number of people that they know who are in the target population. This method has some practical advantages. It does not require direct access to members of the hidden population, and respondents do not reply to any sensitive questions about themselves.^8,9^

 Previous studies on size estimation that calculated the prevalence of drug use in Iran have used direct methods, and their results were prone to underestimation.^10^ In response to the request of the Iranian Ministry of Health and Medical Education in 2013, our research team conducted a national study and provided the size of people who use drugs or alcohol applying the indirect NSU methodology. We found that the annual prevalence of opium and alcohol use were about 1.5% and 2.3%, respectively.^10,11^ To update the size estimates and to address the trends over time, we designed the second phase of the study between 2015 and 2016 using the same methodology.

## Methods

 This cross-sectional study was conducted in all 31 provinces of Iran from October 2015 to March 2016. In each province, the samples were recruited from the capital and from one of the randomly selected cities. The sample size in each province was 500 (half men, half women). The only exception was the Tehran province (the capital of the country), where due to its cultural diversity, we recruited 1000 subjects. Based on information from civil organizations, each city was divided into three strata with different socioeconomic statuses. In each stratum, two to four public places (ie, the main streets, squares, and parks) were selected. Using street-based sampling, participants were recruited from selected venues. Data were collected on all weekdays, in the morning and evening. We adopted street-based sampling, as responses to sensitive topics are more reliable in street-based sampling than phone or home-based surveys in Iran.^12,13^ Interviewers were not allowed to approach people in shopping centers, offices, or workplaces. We matched the gender of interviewers with participants.

 Before the data collection process, verbal consent was obtained from those who agreed to participate.

###  Network Scale-Up Methodology

 In the NSU method, it is assumed that the prevalence of a behavior in the network of a randomly selected sample is similar to that of the general population. This indicates that the estimation of network size (shown by C) is a prime factor for NSU studies.

 To estimate C, we need some reference groups with known sizes (shown by e). Each person’s social network size is calculated by the following formula:


(1) $$c_{i}=\frac{\sum m_{ij}}{\sum e_{j}}*t$$

Here, the indices *i* and *j* refer to the respondent and reference groups, *t* is the size of the general population, and $\sum_{i}m_{ij}$ is the total number of respondents in all the reference groups. The final C was calculated as the average of *Ci* values.

 We have already estimated the average network size (ie, C) of the Iranian population as 308.^14^

 In the current study, we asked respondents to report the total number of people they know who used any one of the following drugs: (*a*) at least once in the past 12 months or (*b*) frequently (defined as two to three times per week). The standard definition of “know” was applied as follows: “people whom you know and who know you, in appearance or by name, with whom you can interact, if needed, and with whom you have had personal contact over the last 2 years by telephone or email.”^14,15^

 The respondents were asked to stratify their reply by age and gender groups.^14,15^ Population size estimations were considered for the following populations: (*a*) any type of illicit drug (traditional or synthetic drugs); (*b*) opium (locally known as “*Teriak*,” which has two forms: “*Sookhteh*” and Shire); (*c*) heroin and/or crack (H-C); (*d*) stimulants including methamphetamine, ice, ecstasy (X pill), cocaine or coke, Ritalin or methylphenidate; (*e*) hallucinogens including lysergic acid diethylamide (LSD), ketamine, and hallucinogenic fungi (ie, mushrooms); (*f*) liquor; and (g) people who inject drugs (PWID).

 We should mention that the components of crack available in Iran are not the same as crack cocaine. Instead of cocaine, Iranian crack mostly contains heroin, codeine, morphine, and caffeine with or without other drugs.^10,16^ Therefore, in this research, we grouped these two types of drugs because of the similarities between heroin and Iranian crack.

 For data cleaning, replies above 30 were rounded to this threshold. Moreover, 0.1% of cases, for whom the difference between “total known” and “summation of men and women known” was higher than two, were excluded from this analysis.

 To estimate the size of people who belonged to a specific population, we applied the following formula:


(2) $$e=\frac{\sum_{i}m_{ij}}{\sum_{i}c_{i}}*t$$

In this equation, *i *and *j* refer to the respondent and hidden groups, respectively, *t* is the size of the general population, $\sum_{i}c_{i}$ is the total network size of respondents estimated by formula (1), and $\sum_{i}m_{ij}$ is the total number of people known by the respondents.^14-16^

###  Visibility Correction Factor

 One of the assumptions of the NSU method is that the respondents are aware of the sensitive behaviors of the members of their network. As sensitive information may not be transmitted, this assumption is hard to meet. In our previous research, by applying the game of contact methodology, we estimated the visibility of drug injection and alcohol consumption at about 54%.^17,18^ Additionally, we assumed that the visibility factor for other types of drugs was slightly lower at 45%. To adjust the NSU method estimates, the crude estimates were divided by the visibility factor.^19^

###  Uncertainty Level

 The bootstrap method was applied to provide 95% uncertainty levels (ULs). We drew 1000 independent samples by replacement from the original data. Using each sample, the prevalence was calculated. Also, the percentiles 2.5% and 97.5% were considered as the lower and upper bounds of UL, respectively.

###  Other Aspects

 We stratified the data by province to provide province-level prevalence estimations. Due to the small sample size, the province-level estimates were smoothed by calculating the weighted average of province-level and national estimates. The weights applied to province-specific and national estimates were four and one, respectively. We applied quartiles to categorize the country into four risk zones. All these analyses were performed in R, Excel, and Arc Map version 9.3.

## Results

###  Study Respondents

 Our final sample comprised of 15 124 respondents equal to 94.5% response rate, of which 54% were men, 35.1% had never married, and 80.5% had high school or university degrees (Table 1). The mean (±standard deviation [SD]) age was 33.8 (±11.2) years old (ranging from 18 to 88 years old). The proportion of respondents in different age groups, marital status categories, and education levels was comparable to that of the general population. Due to the large sample size and sufficient power, we did not statistically compare the difference between proportions.

**Table 1:** Sociodemographic Characteristics of the Participant to Size Estimation of Illicit Drugs and Alcohol in Iran Using Network Scale-Up

**Demographic Characteristics**	**No. (%)**
Gender	
Men	8078 (54)
Women	6868 (46)
Age	
18-30 years	6361 (42.9)
30-50 years	6919 (46.6)
Above 50 years	1566 (10.6)
Marital status	
Never married	5227 (35.1)
Currently married	9125 (61.3)
Previously married (eg, divorced/widowed)	529 (3.6)
Education	
High school or less	8120 (54.5)
Above high school	6767 (45.5)

###  Prevalence Estimates for At-least Once 

 The annual prevalence of “any type of illicit drug use” was 7498 per 100 000 population. The most commonly used illicit drugs were as follows: opium (2534 per 100 000 population), hashish (849 per 100 000 population), stimulants (methamphetamine, ecstasy pills, cocaine, and Ritalin) (842 per 100 000 population), heroin/crack (578 per 100 000 population), injection drugs (459 per 100 000 population), non-prescription methadone (495 per 100 000 population), and hallucinogens (LSD and ketamine) (220 per 100 000 population). Additionally, we estimated the number of people who use alcohol as 2797 per 100 000 population (Table 2).

**Table 2:** The Annual Prevalence and Estimated Size of Various Groups of Drug and Alcohol Users in Iran Using Network Scale-Up

	**At-least Once**			**Frequent Use**^a^		
	**Prevalence**^b^	**Frequency**^c^	**UL for frequency (95%)**	**Prevalence**^b^	**Frequency**^c^	**UL for Frequency (95%)**
Any type of drugs	7498	5 634 530	(5 310 918, 6 003 103)	4791	3 600 301	(3 364 068, 3 866 650)
Alcohol	2797	2 101 818	(2 052 298, 2 150 046)	830	623 784	(594 955, 654 988)
Opium	2534	1 904 244	(1 853 895, 1 952 393)	1780	1 337 983	(1 290 158, 1 380 468)
Hashish	849	637 821	(609 643, 665 662)	461	346 630	(325 877, 367 484)
Heroin or crack	578	434 734	(413 415, 456 270)	408	306 667	(287 852, 325 554)
Simulants	842	632 908	(602 469, 660 623)	492	369 692	(348 286, 390 953)
Hallucinogens	220	165 558	(152 796, 178 611)	104	77 903	(69 693, 86 767)
PWID	459	345 308	(329 068, 363 364)	323	242 651	(227 452, 257 079)
Non-prescription methadone	495	372 138	(350 003, 393 436)	298	224 079	(206 496, 241 704)

Abbreviations: UL, uncertainty level; PWID, people who inject drugs. ^a^Two to three times per week; ^b^Per 100 000 population; ^c^In the total/country population

###  Prevalence Estimates for Frequent Use 

 The frequency of illicit drug use was 4791 per 100 000 population. In other words, 4.79% (95% UL [3 364 068, 3 866 650]) of the Iranian population used drugs frequently. Opium and hallucinogens had the highest and lowest prevalence of usage at 1780 and 104 per 100 000 population, respectively (Table 2). In addition, the frequent prevalence of alcohol consumption was estimated at 830 per 100 000 population.

###  Gender Differences 

**Figure 1:**
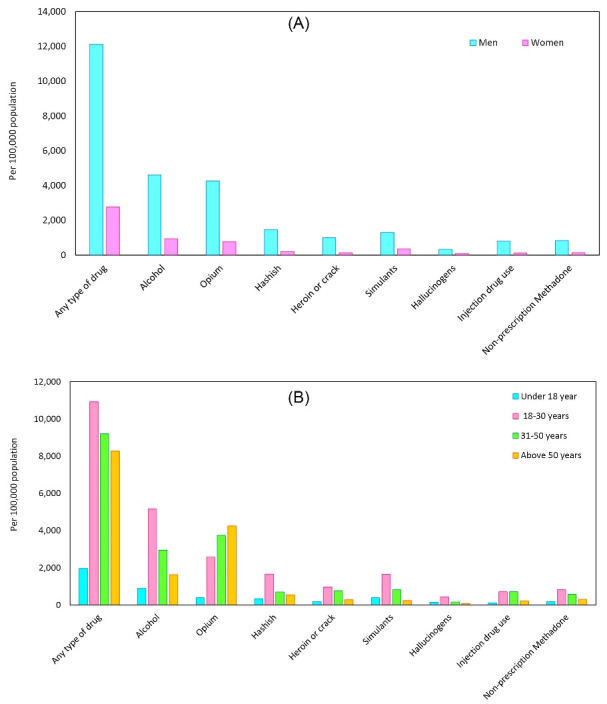
At Least Once the Prevalence of Illicit Drug Use in Iran Across Gender (A) and Age (B) Groups.

 The most popular drugs in both genders were opium, hashish, and stimulants. However, the prevalence among men was much higher than in women (opium: 4271 versus 766 per 100 000 population; hashish: 1476 versus 210 per 100 000 population; and stimulants: 1304 versus 372 per 100 000 population). The large gender differences were seen in other drugs and alcohol. For example, the prevalence of alcohol consumption in men was nearly five times higher than that of women (4613 versus 941 per 100 000 population) (Figure 1).

###  Age Differences

 Among those less than 18 years old, the most popular drugs were opium and stimulants, and they frequently consumed alcohol (388, 387, and 887 per 100 000 population, respectively).

 Drug use was more prevalent among respondents aged between 18 and 50 years old (Figure 1). The most popular drug among those aged above and under 30 years old was opium, and they frequently consumed alcohol.

 The prevalence of opium use was found to be the highest among those aged above 50 years old (4253 per 100 000 population).

 A similar pattern was seen among those aged between 18 and 30 years old. Drug injection was mostly popular among those aged between 31 and 50 years old. However, the highest proportion of hallucinogen use was observed in the subjects aged between 18 and 30 years old (Figure 1).

###  Geographical Variations

**Figure 2:**
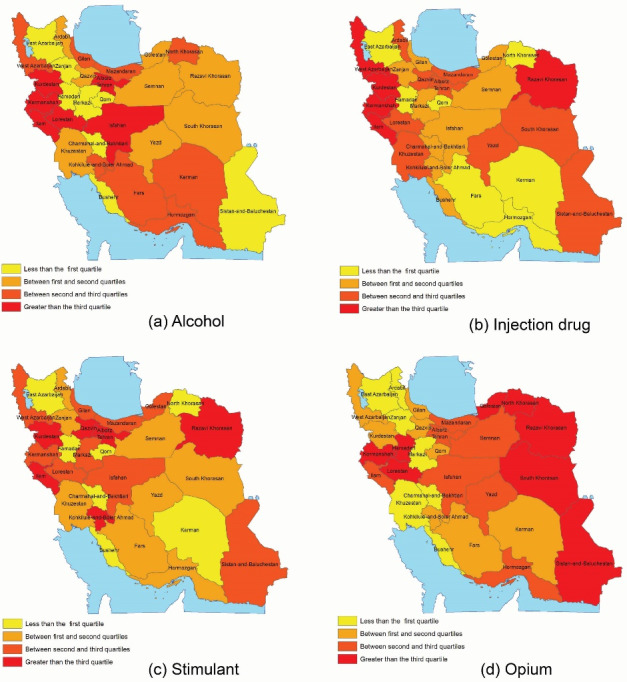
Distribution of the 12-Month Prevalence of Substance Drug Use.

 The prevalence of opium use was found to be higher in the southern and eastern provinces of the country (Figure 2). On the other hand, the high-risk zones were large and industrial provinces in terms of stimulant use. Regarding alcohol consumption, the western and southwestern provinces were categorized into the high-risk zone. Finally, the central and western provinces were the high-risk zones for drug injection.

## Discussion

 Our study was the second phase of a national mission aimed to estimate the prevalence of drug and alcohol use in Iran and to monitor its trend. The first phase was conducted in 2013.

 In phase two, in light of our ongoing experience with the NSU method, we updated our questionnaire and estimated the prevalence of several types of drugs.

 We found that the annual prevalence of “any drug type” among the Iranian population was about 7.5% for “at least once” and 5% for “regular use.” Corresponding estimates for alcohol use were 2.5% and 0.8%, respectively. In 2007, the prevalence of substance use in Iran was reported as 2.4% through a rapid assessment. Furthermore, in 2011, the results of a national household survey showed that approximately 3.0% of Iranians regularly used certain types of substances. Although the comparison of the results of these studies might be misleading due to methodological and measurement differences, these studies may point to an increasing trend in drug use.

 In the current study, after alcohol, the most frequently used drugs in both genders and all age groups were opium and hashish. In line with this finding, the most frequently used drugs were opium and methamphetamine in a study in 2011^20^ and opium, crack, and heroin in a study in 2007.^21^

 In the previous NSU study conducted in 2013, opium and crystal methamphetamine had the highest annual prevalence (at least once). These findings suggested that, over time, PWID tended to use synthetic drugs rather than traditional drugs. This hypothesis has also been confirmed in the findings of some other studies.^6^

 While in 2013, our estimate of PWID was 208 000, our updated figure showed a 66% increase at 345 308.^8^ Based on a Ministry of Health report, there are between 170 000 and 230 000 PWID in Iran.^22^ The increased number of PWID could be related to the changing pattern of drug use in the country. As PWID are still a high-risk group for transmitting HIV and hepatitis C infections in Iran, this increase is alarming.^22^ Due to needle sharing, multiple uses of needles, and risky sexual behaviors, the prevalence of HIV and hepatitis C infections are high among PWID.^23^ An important difficulty with this group is that only a tiny percentage of PWID receive substance use treatment due to the stigma around drug injection.^24,25^ Therefore, special measurements are required for finding cases and providing harm reduction services.

 The current estimates for the prevalence of stimulants and hallucinogens are 0.84% and 0.22%, respectively. In 2013, we estimated the prevalence of amphetamine/ecstasy/LSD and crystal methamphetamine at 0.3% and 0.59%, respectively. In another study, we found that nearly 80% of Iranian youth had low to medium levels of knowledge of methamphetamine. Moreover, we observed that women were more likely to take methamphetamine to lose weight.^26,27^ This highlights the importance of educational packages enhancing the knowledge of the youth about synthetic drugs.

 Our study showed the annual prevalence of alcohol users was 2.7%, of whom 0.8% were using regularly. Our previous estimation in 2013 reported an estimate of 2.3%, which is fairly close to the current figure. The World Health Organization’s (WHO’s) report published in 2016 reported an annual prevalence of 1% among the population aged >15 years old in Iran.^28^ Due to methodological differences, our estimates are not comparable with the estimates of the WHO. However, we believe our estimates may be more accurate, as we applied an indirect method among members of the general population.

 The prevalence of drug and alcohol use among men was higher than among women. For example, alcohol use was five times higher among men compared to women. These differences can be attributed to the fact that, culturally, the level of stigma associated with substance use is much higher for Iranian women.

###  Limitations

 An important limitation of our study was that, in each province, the respondents were selected from the capital and one major city. Therefore, the results may not be generalizable to rural areas. Another limitation was that, in a trade-off between representativeness and accuracy, we decided to adopt street-based sampling. Our experience showed that, in Iran, street-based interviews are optimal for prevalence estimation of sensitive characteristics, which may partially be due to its confidentiality. This method suffers from the fact that not all members of the population have an equal chance of being interviewed. Furthermore, due to the stigma around drug-related behaviors and methodological challenges, the estimated sizes should be interpreted with caution. We recommend triangulating our results with other available data to get a more robust size estimate.

 Moreover, our estimations were based on self-report responses of the respondents on behalf of their network size. Although we applied the correction factors to address the invisibility, the results should be interpreted with caution. Lastly, our estimations were based on data obtained in 2016, so these might not provide a current picture of substance use in Iran. However, these estimations can still be helpful and informative for policy planning and resource allocation. These are the most updated/recent data on population size estimation for substance use at the national level.

## Conclusion

 In this research, we observed an increasing trend in the prevalence of drug use in Iran. Opium was found to be the most frequently used drug in all age and gender groups. Also, drug use was more prevalent among men than among women, which may be due to a higher level of stigma and the current cultural context for the population of women. In terms of age groups, drug use was more prevalent among the young and middle-aged populations aged between 18 and 50 years old, which might be due to the higher economic, social, and emotional pressures experienced by these age groups. Our results suggest that a range of interventions programs should be designed for different age and gender groups.

## Acknowledgements

 The authors are grateful to the provincial interviewers assisting in data collection. We also acknowledge the Ministry of Health and Medical Education for funding this study. MS was supported by the Canadian Institute of Health Research (CIHR). MK was supported by the Pierre Elliott Trudeau Foundation Doctoral Scholarship.

## Ethical issues

 We explained the aim of the study to the participants, and only those who verbally agreed to fill in the questionnaires were recruited. The ethics committee of Kerman University of Medical Sciences has approved the research protocol. The code number and ethical approvals were 910208 and 163/90/KA, respectively.

## Competing interests

 Authors declare that they have no competing interests.

## Authors’ contributions

 All authors contributed to the study conception and design. Data collection was performed by AR. Data analysis was performed by AR, MRB, and all authors contributed to interpretation. The first draft of the manuscript was written by AR, AH, and MRB and all authors commented on previous versions of the manuscript. All authors read and approved the final manuscript.

## Funding

 This work was supported by the Ministry of Health and Medical Education, Tehran, Iran. This work has been granted by the Ministry of Health and Medical Education (grant number: 97000071).
